# Draft Genome Sequence of the Vaginal Isolate *Corynebacterium amycolatum* ICIS 9

**DOI:** 10.1128/genomeA.00975-17

**Published:** 2017-09-14

**Authors:** Irina V. Gladysheva, Yuriy A. Khlopko, Sergey V. Cherkasov

**Affiliations:** Institute for Cellular and Intracellular Symbiosis, Ural Branch of Russian Academy of Sciences, Orenburg, Russia

## Abstract

*Corynebacterium amycolatum* ICIS 9 was isolated from a vaginal smear of a healthy woman. Here, we report the draft genome sequence of *C. amycolatum* ICIS 9, which will be useful for further studies of specific genetic features of this strain and for understanding its probiotic properties.

## GENOME ANNOUNCEMENT

Vaginal microbiocenosis is an open complex multicomponent system that exists in a state of dynamic equilibrium. It is widely known that lactobacilli play a major role in protecting the vaginal environment from nonindigenous and potentially harmful microorganisms (1, 2). Using culture-independent techniques, several investigators have demonstrated that a significant proportion (7 to 33%) of healthy women lacks appreciable numbers of *Lactobacillus* spp. in the vagina (3, 4), which may be replaced by other lactic acid-producing bacteria such as *Atopobium vaginae* and *Megasphaera*, *Leptotrichia*, and *Corynebacterium* spp. (5). Therefore, in the absence of lactobacilli, these organisms are probably involved in protecting the vaginal biotope from infection.

We carried out a pilot study to determine the biological properties of vaginal isolates of microorganisms of the genus *Corynebacterium*. We found that *Corynebacterium* spp. that are isolated from healthy women have potential probiotic properties. Metabolites of these strains significantly increased antagonistic activity of the peroxide-producing lactobacilli (6), inhibited growth and biofilm formation, and destroyed 1-day-old (24-h-grown) pathogenic and opportunistic microorganisms (7). Here, we report the draft genome sequence of *Corynebacterium amycolatum* ICIS 9, which will be useful for further studies of specific genetic features of this strain and for understanding its probiotic properties.

The strain was grown in tryptic soy broth (Sigma-Aldrich Co., USA) at 37°C. Genomic DNA was extracted from an overnight culture using the phenol-chloroform method. The preparation of DNA libraries and sequencing were carried out at the Center of Shared Equipment “Persistence of Microorganisms” at the Institute for Cellular and Intracellular Symbiosis, UrB RAS (Orenburg, Russia). Library preparation was performed using the Nextera XT DNA library preparation kit (Illumina, USA) according to the manufacturer’s instructions. Sequencing was performed on an Illumina MiSeq platform using a MiSeq version 3 reagent kit (Illumina). The reads were quality trimmed using the sliding window mode of the Trimmomatic program (8). *De novo* genome assembly was performed using a SPAdes genome assembler (St. Petersburg genome assembler version 3.9.0) (9). The draft sequence consists of 181 contigs of more than 200 bp, with an average coverage of 22.0×, an *N*_50_ length of 45,496 bp, and an *L*_50_ value of 18 bp. It has a G+C content of 58.67% and a total length of 2,587,830 bp. Genome annotation was performed using the NCBI Prokaryotic Genome Annotation Pipeline (http://www.ncbi.nlm.nih.gov/genome/annotation_prok), which identified 2,392 coding sequences, including 2,330 proteins, 53 pseudogenes, and 6 rRNAs (3 complete rRNAs [5S, 16S, and 23S] and 3 partial rRNAs [5S]).

Genome sequence analysis of *C. amycolatum* ICIS 9 showed the presence of genes that are responsible for the biosynthesis of vitamins (biotin, riboflavin, and pyridoxine), folate, lipoic acid, and other cofactors; a large number of genes of stress response systems, including those related to oxidative stress, heat shock, and cold shock; and a number of genes that contribute to antibiotic resistance (vancomycin, fluoroquinolones, tetracycline, and beta-lactam) and heavy-metal resistance (arsenic, mercury, and cobalt-zinc-cadmium). A number of genes are responsible for the synthesis of possible biosurfactants, such as phospholipids, triacylglycerols, fatty acids, and neutral lipids.

### Accession number(s).

This whole-genome shotgun project has been deposited at DDBJ/ENA/GenBank under the accession number MTPT00000000. The version described in this paper is the first version, MTPT01000000.
